# Comparison of selected cryoprotective agents to stabilize meiotic spindles of human oocytes during cooling

**Published:** 2010-10-04

**Authors:** Dunsong Yang, Kevin L. Winslow, Kevin Nguyen, Daniel Duffy, Michael Freeman, Talha Al-Shawaf

**Affiliations:** 1 Florida Institute for Reproductive Medicine, Baptist Medical Center Pavilion, Jacksonville, Florida, USA; 2 Barts and The London Centre for Reproductive Medicine, St. Bartholomew’s Hospital, West Smithfield, London, UK

**Keywords:** cryoprotective agents, spindle, cryopreservation, human oocyte, cooling, Taxol

## Abstract

**Background:**

This study examined the primary effect of selected cryoprotective agents (CPAs) on the meiotic spindles of human oocytes during cooling.

**Methods:**

Fresh metaphase II oocytes (*n*=26) donated from patients undergoing IVF treatment were analyzed via Polscope. In experiment one, 16 oocytes with visible spindle at 37°C were cooled to 20°C and rewarmed to 37°C to test the spindle response to cooling. They were then cooled to 20°C, 10°C, 0°C and rewarmed to 37°C after having been equilibrated with 1.5 M 1,2-propanediol (PROH), 1.5 M dimethyl sulfoxide (DMSO), 1.5 M ethylene glycol (EG) or 10 μM taxol at 37°C. In experiment two, 10 oocytes without visible spindles at 37°C were cooled to 20°C and then equilibrated with PROH, EG and taxol at 20°C. Spindle images were recorded at each temperature.

**Results:**

Meiotic spindles remained visible or became more distinct during cooling to 20°C, 10°C and 0°C when equilibrated with PROH, EG, DMSO and Taxol. Without these agents, meiotic spindles of the same oocytes disappeared after cooling to 20°C.

**Conclusion:**

The primary effect of PROH, EG and DMSO on the meiotic spindle is to stabilize and protect it against low temperature disassembly. A higher equilibration temperature (≥33°C) for oocyte freezing is recommended.

## Introduction

Studies on human oocyte cryopreservation over the last decade yielded only limited success, while human embryo cryopreservation has been performed successfully around the world for many years. The suboptimal results in oocyte cryopreservation may be a result of damage to meiotic spindle during freezing and thawing. The meiotic spindles in mammalian oocytes, including the human, are extremely sensitive to temperature change [1–4]. Exposure of oocytes to room temperature (25°C) or lower for as little as 1–5 min has been shown to cause depolymerization of the meiotic spindle [4,5]. Cryoprotective agents (CPAs) are essential components in freezing solutions, but may also disrupt the integrity of meiotic spindle. Vincent and colleagues [6,7] found that exposure of mouse oocytes to 1.5M DMSO and PROH resulted in microfilament depolymerization, and the absence of meiotic spindle in oocytes after cryopreservation was attributed to the toxic effect of cryoprotectants [8,9].

Due to concerns about toxicity of CPAs, oocytes are normally cooled to room temperature (18°C to 24°C) or even lower (4°C to 0°C) before equilibrating with CPAs [10–14]. However, CPAs may have protective effects on the meiotic spindle, and therefore should be applied prior to cooling. Previous studies have shown the ability of both DMSO [15,16] and glycerol [17–19] to enhance formation of microtubules. Both DMSO and PROH have been reported to have protective effects on the meiotic spindle of mouse and human oocytes [20,21]. Several recent investigations [22–28] incorporating traditional fixation and immunostaining techniques have raised questions regarding the protective effects of CPAs. Profound spindle alterations and spindle disassembly in oocytes after cryopreservation were displayed immediately upon thawing [23,26,27], although partial spindle recovery may be achieved after 1–3h of incubation [24]. In contrast, studies using a computer-assisted polarization microscopy system (Polscope) have provided compelling evidence for a protective effect [29,30]. Oocyte spindles were identified during room-temperature equilibration with PROH and sucrose as cryoprotectants and immediately after thawing; spindles disappeared with subsequent removal of cryoprotectants and reappeared after 1–3h of culture [29]. Ice formation and excessive dehydration during oocyte freezing and thawing may also damage spindles and oocyte chromatin [31,32]. It remains unclear if disappearance of spindles after thawing results from a toxic effect of cryoprotectants and/or the physical stress of the freeze-thaw sequence. Understanding the primary effects of CPAs on the meiotic spindles is therefore essential in developing improved cryopreservation protocols.

The present research using a Polscope system and a cooling stage attached to the same microscope was designed to assess the impact of commonly used penetrating CPAs, (*i.e*., PROH, EG and DMSO) on meiotic spindles of human oocytes by cooling (without freezing) and re-warming the same oocytes with and without one of the cryoprotectants. Taxol, a known spindle stabilizing agent was tested as a reference reagent. Based on these findings, a higher equilibration temperature (≥33°C) for oocyte freezing is recommended.

## Materials and methods

### Oocyte procurement and reagents

Twelve patients (four of whom were oocyte donors) provided a total of 50 oocytes for this study. Eight of them who had 20 to 40 oocytes (mean 30.6 oocytes) available for IVF donated 3 to 5 oocytes each, two oocyte donors underwent split cycles without a second recipient donated half of the retrieved oocytes, and two patients who chose to inseminate limited number of oocytes for transfer donated half of their oocytes. Mean (±SD) age of patients was 30.58 ± 9.44 years. After screening oocytes for visible spindles using Polscope, all oocytes without visible spindle (*n*=10) and 16 of the 40 oocytes with spindle were randomly selected for study. The remaining oocytes were discarded because only a maximum of 3 oocytes from the same patient could be analyzed within 40–44h after hCG injection. The study was approved by the Institutional Review Board (IRB) of Baptist Medical Center, Jacksonville, Florida and written informed consent was obtained from all study subjects.

Controlled ovarian hyperstimulation was preceded by pituitary downregulation with GnRH agonist (Lupron; TAP, Deerfield, IL), followed by recombinant FSH (Follistim; Organon, Roseland, NJ) and hMG (Pergonal; Serono). 10,000IU hCG was used for triggering. Transvaginal oocyte aspiration was performed under i.v. sedation 35h after hCG injection. Following retrieval, oocytes were stripped of cumulus cells after being cultured in IVC-ONE medium (InVitroCare, Frederick, MD) supplemented with serum substitute (SSS; Irvine Scientific, Santa Ana, CA) for 2–4h.

Unless stated otherwise, all reagents were purchased from Sigma Chemical Company (St. Louis, MO, USA). All oocyte treatment solutions were prepared using Dulbecco’s phosphate-buffered solution (PBS) (Sage Biopharma, Bedminster, NJ) and contained a final concentration of 20% SSS (Irvine Scientific). Treatment solutions used were: (1)1.5M 1,2-propanediol (PROH); (2) 1.5M dimethyl sulfoxide (DMSO); (3) 1.5M ethylene glycol (EG) and (4) 10μM taxol.

### Spindle examination

For spindle imaging, each oocyte was placed in10μl of treatment solution covered with mineral oil (Medicult, Denmark) in a glass-bottomed culture dish (Willco Wells, Amsterdam, The Netherlands). An inverted microscope equipped with a Peltier system (PE100, Linkam Scientific Instruments Ltd, UK) was used for oocyte assessment and spindle examination. The Peltier system includes a biological warming and cooling microscope stage attached to a temperature controller with a range of −5°C to 99°C (PE-94, Linkam Scientific Instruments Ltd). Dishes were placed on the stage during equilibration and examination. During examination, oocytes were manipulated by holding pipette (Sunlight Medical, Jacksonville, FL) and/or partial zona dissection pipette (Sunlight Medical). The meiotic spindle visualization was performed at 200X magnification with LC Polscope optics and controller (SpindleView; CRI, Woburn, MA, USA) fitted to a computerized image analysis system (SpindleView software; CRI).

### Experimental designs

**Figure 1.**
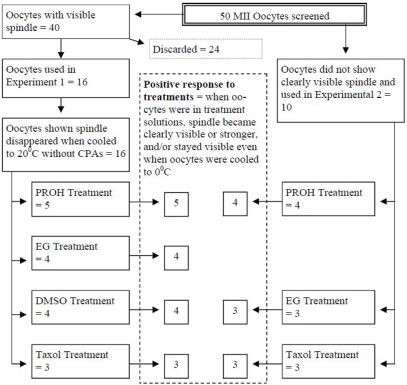
Oocyte allocation for Experiments 1 and 2 with summary of response to selected cryoprotectants.

Details of the oocyte spindle status, experimental assignment and treatment subgroups are summarized in Figure 1. Oocytes were first placed in a drop of PBS in a glass-bottom dish at 37°C on the microscope stage. Spindle images were recorded and oocytes were placed in two study groups as a function of spindle visualization as follows: If a clear oocyte spindle was visible, oocytes were entered into Experiment 1; if no spindle was noted, then oocytes were used for Experiment 2. As stated previously, only 16 of 40 MII oocytes with a visible spindle were actually used in Experiment 1.

### Experiment 1

Oocytes in Experiment 1 were cooled to 20°C at −5°C per minute, held for 5min and then rewarmed to 37°C at 5°C per minute, to measure spindle response to temperature change. Oocytes showing a positive response to temperature change (*i.e*., spindle loss at 20°C and recovery after rewarming to 37°C) were continued in Experiment 1. Spindle images were recorded after reaching the desired temperature and at 5min. These images were used as controls for comparison with treatment by the different chemical agents.

Oocytes were then exposed to one of four treatment solutions at 37°C x 10min. The stage temperature was then dropped to and held for 10min at 20°C, 10°C, 0°C and then rewarmed to 37°C. Spindle images were taken 5 and 10min after reaching the desired temperatures. The experiment was repeated on at least three oocytes for each agent.

### Experiment 2

Oocytes without visible spindle at 37°C in PBS were cooled to 20°C and then exposed to PROH, EG and taxol at 20°C x 10min. Polscope images were taken before treatments, at 5 and 10 min after equilibration in each treatment solution at 20°C. Cryoprotectants were removed by washing oocytes in PBS three times. The oocytes were then maintained in PBS at 20°C for 10 min before rewarming to 37°C. Next, oocytes were cultured 18–20h in our embryo culture medium (IVC-ONE supplemented with 5% SSS) at 37°C. Polscope images were recorded 5 and 10min after removal of cryoprotectants at 20°C, after 30 minutes in culture at 37°C, and after overnight culture before discarding. The experiment was repeated on at least three oocytes for each agent. DMSO was omitted from this experiment since it induced an extra spindle structure in 2 of 4 treated oocytes. It was our concern that DMSO may not be a suitable human oocyte freezing reagent given this finding.

## Results

Oocyte assignment to each treatment group, responses to temperature modifications and various cryoprotectants are illustrated in Figure 1. All oocytes in both experiments showed positive responses to temperature change and treatment reagents.

### Experiment 1

**Figure 2.**
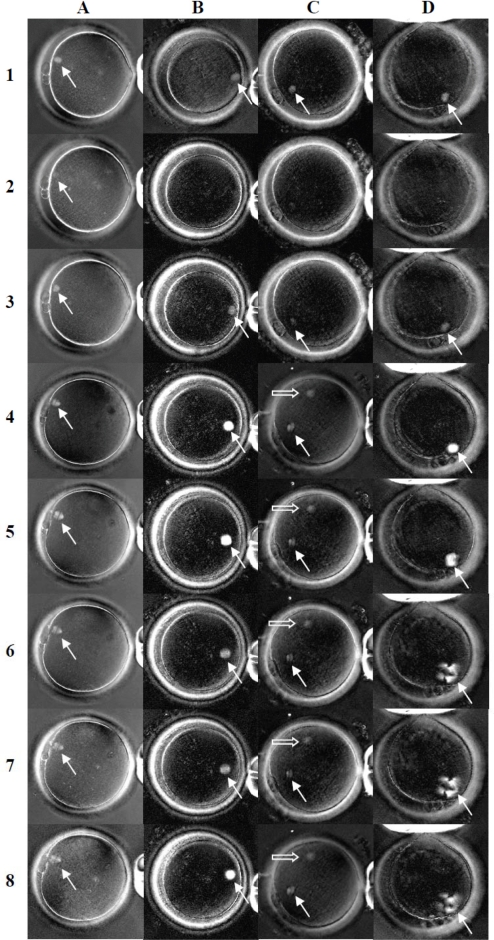
Spindle images of human oocytes following cooling and treatment with cryoprotective agents (Experiment 1). Images in column A, B, C, and D (representing four treatment groups, PROH, EG, DMSO and taxol respectively) were taken after oocytes were maintained in PBS at 37°C (row 1), after temperature dropped to 20°C (row 2), then rewarmed to 37°C (row 3), after having been equilibrated with the cryoprotective agents at 37°C (row 4), after temperature dropped to 20°C (row 5), 10°C (row 6), 0°C (row 7) and then rewarmed to 37°C (row 8). Arrow outlines show spindles while solid arrows in column C indicate newly formed extra spindle structures [original magnification = 200X].

Figure 2 shows spindle dynamics after cooling and treatment with selected cryoprotectants. All oocytes showed dynamic changes in response to cooling from 37°C to 20°C and then rewarming to 37°C in PBS. Spindles which were visible in all oocytes at 37°C (Fig. 2, row 1) became faint (Fig. 2, A2), or disappeared entirely (B2, C2 and D2), at 20°C. Spindles recovered 5min (Fig. 2, row 3) after being warmed to 37°C.

The spindle dynamics in response to temperature change without any CPA served as a control for each oocyte group. The oocytes were then equilibrated with one of the four treatment solutions (PROH, EG, DMSO and taxol) at 37°C. Within 5min after oocyte equilibration in each reagent, the spindle images became larger (A4, C4) and/or more intense (B4, D4) than in PBS. Two of the four oocytes treated with DMSO developed an extra spindle structure (C4). Oocyte spindles in all four treatment solutions were clearly visible after the temperature was dropped to 20°C (row 5), 10°C (row 6) and even 0°C (row 7). Multiple irregular spindles were observed in the taxol group at temperatures down to 0°C (D6, D7), and this formation continued following oocyte rewarming to 37°C (D8). Spindle images of oocytes in EG became weaker at 10°C (B6) and 0°C (B7) compared to 20°C (B5) and 37°C (B8), while in PROH and DMSO spindles showed no obvious changes at 20°C (A5, C5), 10°C (A6, C6), 0°C (A7, C7) or after warming to 37°C (A8, C8) (including extra spindle structure in the DMSO treated oocyte).

### Experiment 2

Spindle dynamics of human oocytes equilibrated with PROH, EG and taxol at 20°C (and their response to cooling and removal of the protective compounds) were studied in Experiment 2. None of the oocytes showed a spindle structure after being cooled to 20°C before equilibration with one of the three treatment solutions. Spindles became clearly visible 5min after being placed in each treatment solution (row 2). The spindles disappeared within 5min following removal of PROH (P3) and EG (E3), but not in the oocyte treated with taxol (T3). Spindle formation continued even after taxol was washed out and cultured in PBS at 37°C for 30 minutes (T3). Spindles in oocytes treated with EG (E4) and taxol (T4) appeared normal after overnight culture. No spindle could be identified in two of the four oocytes treated with PROH after overnight culture (P4). Weak spindle images were seen in the other two oocytes treated with PROH.

## Discussion

**Figure 3.**
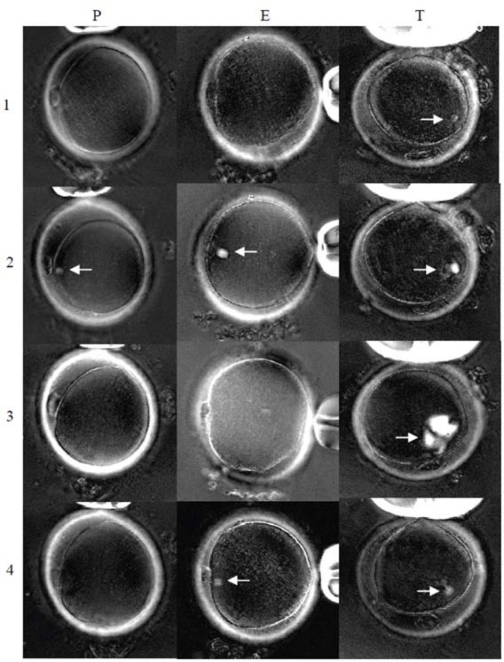
Spindle images of human oocytes equilibrated with cryoprotective agents at 20°C (Experiment 2). Polscope images of oocytes in PROH, EG and taxol groups (Column P = PROH, E = EG and T= taxol) were recorded at 37°C in PBS (row 1), after equilibration with cryoprotective agents at 20°C (row 2), after cryoprotective agents were removed (row 3), and after being cultured overnight at 37°C (row 4). Spindles are designated by white arrows [original magnification = 200X].

Cryoprotective agents have been reported to have beneficial effects on the meiotic spindle of mouse and human oocytes [20,21]. However, some recent studies [22–28] using traditional fixation and immunostaining technique, provide limited support on the protective effects of CPAs on meiotic spindle. On the other hand, cryoprotective agents used in freezing solutions have been regarded as toxic and may disrupt the integrity of meiotic spindle by other investigators [6–9]. Such contradictory findings have complicated the understanding of the effect of CPAs on meiotic spindle. More recent research using a computer-assisted polarization microscopy system (Polscope) [29,30] has showed visible spindles in oocytes during equilibration with PROH and sucrose at room temperature and immediately after thawing, but the spindles disappeared during the subsequent removal of cryoprotectants. Ice formation and excessive dehydration during freezing and thawing may also cause damage to spindles and chromosomes in oocytes [31,32]. It remains unclear if spindle disappearance after thawing results from toxic effects of cryoprotectants and/or the physiologic stress associated with the freeze/thaw sequence. To eliminate the possibility of freezing and dehydration damage to the spindle, the present study analyzed the dynamic change of spindle during cooling up to 0°C with and without the presence of PROH, EG, DMSO or taxol. We found meiotic spindles of human oocyte were visible or became more intense when cooled to 20°C, 10°C and 0°C after equilibrating with each of the tested CPAs at 37°C (Fig. 2). Without CPAs, spindles of control oocytes disappeared after cooling to 20°C. We have demonstrated the ability of PROH, EG and DMSO to stabilize and protect the meiotic spindle against cold induced depolymerization. Interestingly, in all oocytes that did not initially show spindles at 37°C, spindles did become detectable after exposure to PROH, and EG (Fig. 3). It is possible that dynamics of the meiotic spindles in such oocytes (even at 37°C) favored depolymerization. The exposure of the oocytes to PROH and EG may reverse the depolymerization and make spindles detectable, and supports the notion of a stabilizing effect of PROH and EG on meiotic spindle.

Rienzi *et al* [29] reported that spindles not only remained detectable in all oocytes during equilibration with a freezing solution containing PROH and sucrose at room temperature, but became even more intense. These investigators could not identify the protective component in their cryopreservation media, however. Because sucrose is a non-permeable agent, it is possible that PROH is the protective element. The beneficial effects of PROH and DMSO on oocyte cooling and cryopreservation have been reported previously in oocytes. Specifically, Van de Elst *et al* [20] and Gook *et al* [21] found a significantly greater portion of oocytes with normal spindles in the presence of DMSO and PROH than without these reagents, suggesting a protective effect of PROH and DMSO.

Rienzi *et al* [29] reported that the spindle of the surviving oocytes disappeared almost immediately after PROH removal. Spindles reappeared in all oocytes within 3h of culture. Analyzing mouse thawed oocytes after vitrification also showed disappearance of spindles after cryoprotectant (EG) was removed [33]; recovery of spindles was documented in 75% of surviving oocytes. In this study, disappearance of spindles was also recorded after PROH and EG were removed even when oocytes were not frozen or dehydrated (Fig. 3), ruling out the possible spindle damage caused by ice formation and dehydration [32]. In contrast to taxol, which showed continued growth of the spindle after the chemical was removed (Experiment 2), the disappearance of spindle immediately after removal of PROH and EG is reassuring in terms of continued normal oocyte development.

The disappearance of spindle after thaw is of clinical concern. Embryos and oocytes are normally thawed at room temperature and cultured through a sequence of media to remove CPAs before they are cultured in an incubator at 37°C or transferred. The meiotic spindle is a dynamic structure of cytoskeletal filaments, undergoing rapid structural reorganization including filament disassembly at one site and reassembly at another [34]. It is conceivable that without the stabilizing effect of CPAs during and after thawing and when the reassembling activities are not recovered, an accelerated disassembly of spindle microtubules may occur. Although the spindle can be recovered after 1–3h of culture at 37°C in some thawed oocytes, loss of spindle bipolarity and chromosome alignment has been reported [24]. Loss of spindle bipolarity and chromosome alignment may contribute to the poor pregnancy and high miscarriage rates (21–38%) as reported by others [11,35–37]. Prevention of oocyte spindle disassembly during thaw and removal of CPAs could therefore represent an important clinical advance.

It is common practice to cool oocytes to room temperature (18–24°C) [10,11,35] or even lower (0–4°C) [12–14] before equilibrating with CPAs because of concerns over CAP toxicity. This may subject the spindle to a cycle of disassembly and recovery thus altering spindle and chromosome configuration, and ultimately resulting in poor pregnancy outcome.

Reports on CPA effects on the microfilament system are varied, and may be influenced by reagent concentration [38,39], temperature [40], cell type and species [6,41]. At low concentration (1M), PROH was associated with meiotic spindle disruption in mouse oocytes, although it had a stabilizing effect at 1.5 and 2.0 M [38]. Chen *et al* [42] found that the spindles of mouse oocytes disorganized or disappeared when exposed to 5.5M ethylene glycol. Vincent *et al* [7] demonstrated a depolymerizing effect of PROH on actin filaments of early stage rabbit embryos, and even suggested that the actin depolymerization might be one of the reasons for the efficiency of PROH. Exposure of oocytes to 1.5M DMSO caused depolymerization of microtubules in mouse oocytes [6], but showed stabilizing effects on human oocyte spindles during cooling [41]. It is possible that these opposing effects of CPAs on the microfilament system may be influenced by the immunocytochemical technique, i.e., washing, fixation and labeling [7,20] and possible variations among individual technicians. The status of the dynamic structure of the microtubules may be changed during the process, and then captured at the moment of fixation. The advantage of Polscope imaging is the ability to observe the complete range of spindle dynamics in the course of in vitro treatment in a noninvasive fashion, eliminating any question of artifact introduced by the immunocytochemical technique. Using the Polscope, we have demonstrated that the primary effect of PROH and EG on meiotic spindle is to stabilize and protect it from depolymerization. We consider the depolymerizing effect reported by Vincent *et al* [6,43] after thawing and removal of CPA as a “withdrawal effect” of the reagent.

Besides stabilizing the microfilament system, DMSO was associated with the formation of an extra spindle, suggesting a direct or indirect ability to promote polymerization. DMSO has been reported to form multiple cytoplasmic microtubular asters in mouse oocytes [44] as well as promoting rapid formation of microtubules [45] which may favor development of abnormal spindle structures. However, many oocyte vitrification protocols include DMSO as CPA [46–48] with no increased risk of abnormality [46]. This may be helped by the fact that vitrification involves a very short time culture of oocytes with CPAs at low temperature before they are vitrified and that the DMSO is removed right after thawing. A long period of contact with DMSO at physical temperature should be avoided.

The protective effect of CPAs is poorly understood. It has been postulated that CPAs may act by reducing the concentration of intracellular electrolytes, by stabilizing plasma membrane by electrostatic interactions, and by reducing the rates of ice nucleation and crystal growth by increasing the viscosity of extra- and intracellular solutions [49]. Surprisingly little is known about their mode of action at the molecular level. Doebbler and Rinfret [50] observed some correlation between hydrogen bonding capacities of some cryoprotective agents (*i.e*., glycerol, EG) and their protective capacities, suggesting that perhaps the ability to bind or to substitute for water might be an important factor. Hydrogen bonding sites may serve not only to bind water but also in forming and stabilizing an extended region of oriented water around each molecule. The H-bound water oriented around protective solute molecules may be less capable of participating in ice crystal formation. This mechanism may also be responsible for stabilizing the meiotic spindle against cooling. It is our hypothesis that the H-bonding sites of the CPAs may form multiple H-bonds between microtubule and CPA molecules to increase the stability of the microtubule against cold induced depolymerization.

Taxol, an antitumor drug [51], was tested as a reference reagent because it is known to enhance microtubule assembly through a combination of elongation of existing microtubules and spontaneous nucleation of new microtubules in vitro [52,53]. Microtubules polymerized in the presence of taxol are resistant to depolymerization by cold (4°C) [54]. In our study, Polscope imaging demonstrated rapid growth of the spindle structure with multiple poles extending radially, even at 0°C (Fig. 1) and for at least 30min after reagent removal (Fig. 3). Although taxol has been shown to improve preimplantation development of cryopreserved mouse [55], porcine [56], bovine [57] and human [58] oocytes, we believe it should not be used clinically because of abnormal microtubule formation. Further studies may be needed to confirm the finding.

This study examined spindle responses to cooling up to 0°C in presence of selected CPAs. It may not represent spindle response to freezing at −196°C when a few CPAs are often used in clinical oocyte cryopreservation. The reason for the weak or no spindle formation among oocytes treated with PROH (compared to EG and taxol treated oocytes) after overnight culture (Experiment 2) is unknown. More research is needed to identify the cause(s) of such spindle loss.

The present study demonstrates the ability of CPAs to stabilize and protect the meiotic spindle of human oocytes against cooling. It is recommended that oocytes should always be equilibrated with freezing solutions at physiological temperature (37°C), or at least 33°C [4] before cooling. Our preliminary experiments included equilibrating human unfertilized oocytes with PROH at physiological temperature (37°C), and showed improved oocyte survival [59]. The fertilization, survival, embryo development, and pregnancy rates were comparable to fresh oocytes and cryopreserved embryos [60]. The benefits of higher pre-equilibration temperature on oocyte cryopreservation outcome have also been reported by Keskintepe *et al* using vitrification [48]. We believe oocytes should never be cooled without prior protection.
